# Triazole Resistance in *Aspergillus* spp.: A Worldwide Problem?

**DOI:** 10.3390/jof2030021

**Published:** 2016-07-04

**Authors:** Olga Rivero-Menendez, Ana Alastruey-Izquierdo, Emilia Mellado, Manuel Cuenca-Estrella

**Affiliations:** 1 Mycology Reference Laboratory, National Centre for Microbiology, Instituto de Salud Carlos III., Carretera de Majadahonda a Pozuelo Km. 2, Majadahonda, 28220 Madrid, Spain; orivero@isciii.es (O.R.-M.); emellado@isciii.es (E.M.); mcuenca-estrella@isciii.es (M.-C.E.); 2 Spanish Network for Research in Infectious Diseases (REIPI RD12/0015)—co-financed by European Development Regional Fund “A way to achieve Europe” ERDF, Madrid, Spain

**Keywords:** *Aspergillus fumigatus*, aspergillosis, azole drug resistance, *cyp*51A, mutations

## Abstract

Since the first description of an azole-resistant *A. fumigatus* strain in 1997, there has been an increasing number of papers describing the emergence of azole resistance. Firstly reported in the USA and soon after in Europe, it has now been described worldwide, challenging the management of human aspergillosis. The main mechanism of resistance is the modification of the azole target enzyme: 14-α sterol demethylase, encoded by the *cyp*51A gene; although recently, other resistance mechanisms have also been implicated. In addition, a shift in the epidemiology has been noted with other *Aspergillus* species (mostly azole resistant) increasingly being reported as causative agents of human disease. This paper reviews the current situation of *Aspergillus* azole resistance and its implications in the clinical setting.

## 1. Introduction

Invasive aspergillosis (IA) is a life-threatening infection caused by ubiquitous saprophytic *Aspergillus* species, which are the most common cause of invasive mold infections worldwide, especially in immunocompromised patients [1]. *Aspergillus fumigatus* is the leading agent of IA [2] but also of all other forms of aspergillosis, including allergic bronchopulmonary aspergillosis (ABPA), chronic pulmonary aspergillosis (CPA) and aspergilloma [3]. This fungus produces billions of airborne conidia due to an abundant asexual reproduction cycle and has the ability of surviving in very different environments, such as those with temperatures up to 60 °C [4].

Despite the mortality and morbidity of IA remaining high due mainly to difficulties in early diagnosis, the survival rates of these patients have improved due to advances in diagnostics and treatment. The triazoles, itraconazole (ITC), voriconazole (VRC) and posaconazole (POS), are the mainstay of treatment for aspergillosis. Isavuconazole is a new extended-spectrum triazole, and its activity against *Aspergillus* has been proven [5]. Triazoles are the only anti-*Aspergillus* agents that are orally available, making them essential for long-term therapy [6]. Although VRC is recommended as first-line therapy for IA [7,8], ITC is still commonly used for chronic and allergic non-invasive forms of aspergillosis [8,9], and POS was shown to reduce the number of invasive fungal infections in neutropenic patients [10]. Additionally, there are some alternative therapies to triazoles that can function as rescue treatments, such as echinocandins or amphotericin B [8].

## 2. Antifungal Susceptibility Testing and Azole Resistance within *Aspergillus fumigatus*

The Clinical and Laboratory Standards Institute (CLSI) and European Committee on Antimicrobial Susceptibility Testing (EUCAST) have developed reference methods to test antifungal susceptibility, which allow the detection of in vitro resistance nowadays. Both committees defined wild-type (WT) MIC (minimum inhibitory concentration) distributions in order to establish epidemiologic cutoff values (ECVs) for *A. fumigatus* and azoles [11,12]. Based on these data and taking into account the clinical outcome, pharmacokinetics and pharmacodynamics, EUCAST defined breakpoints for *A. fumigatus* and azoles (ITC > 2 µg/mL, VRC > 2 µg/mL, POS > 0.25 µg/mL and ISA > 1 µg/mL), which are used to categorize *A. fumigatus* strains as susceptible or resistant [13]. CLSI has also defined ECVs for *A. fumigatus* and azoles: ITC > 1 µg/mL, VRC > 1 µg/mL, POS > 0.5 µg/mL [11]. There are other commercial methods for in vitro susceptibility testing, such as Etest (BioMerieux, Marcy l’Etoile, France) or Sensititre YeastOne (SYO) (Trek Diagnostic Systems Ltd., East Grinstead, UK), that are complementary to EUCAST and CLSI and are easy to perform for routine use.

Since the first reported case in 1997 in clinical *A. fumigatus* isolates collected in the 1980s in the U.S. [14], an ever-growing number of triazole resistance strains have been published [1,6]. The increased description of azole-resistant *A. fumigatus* strains in the last few years may pose a threat to public health because of the lack of alternative treatment [15,16]. In addition, as in vitro antifungal susceptibility testing in *Aspergillus* is not routinely done in non-invasive settings, the prevalence of triazole resistance strains is likely to be underestimated [6].

## 3. Azole Resistance Development in *Aspergillus fumigatus*

*Aspergillus fumigatus* is usually susceptible to azoles, but as stated before, secondary resistance is increasingly reported. Since the first azole-resistant isolate detected in 1997 in the U.S., azole resistance has been increasingly reported from many other countries. Particularly in the past few years, there has been an increase in clinical resistant isolates described from the Netherlands [6]. This is well studied at the molecular level and will be further discussed in this review. The development of secondary resistance is thought to be acquired in two possible ways. In patients that suffer chronic aspergillosis and are under long-term azole treatment, resistance can develop through this exposure [17]. These patients are initially infected by a susceptible *A. fumigatus* strain that evolves to a resistant phenotype under azole treatment pressure. These resistant isolates are isogenic to the initial one that caused the infection. Camps et al. reviewed seven cases of acquired resistance during treatment showing an average delay of four months between the latest susceptible and the first resistant isolate [18]. The first reported case of this resistance route was described in 2001 in four isogenic *A. fumigatus* isolates recovered from a patient treated with ITC for a pulmonary *A. fumigatus* infection. Two of them were obtained before treatment with ITC, and two were isolated after treatment finished. The results suggested that the strain acquired resistance to this antifungal during treatment [19]. Alternatively, the use of azole fungicides in the environment that induce cross-resistance to medical triazoles in environmental *A. fumigatus* isolates has been suggested as another source of resistance development [20]. This environmental route was described in a study were a single mechanism of azole resistance was found in 94% of clinical isolates from several hospitals in The Netherlands [20], not being able to relate it to previous antifungal treatment. Finally, intrinsic azole resistance has also been described in other *Aspergillus* spp.

## 4. Mechanism of Azole Resistance

Since the first report of the *A. fumigatus* azole resistance strain, several studies have been published investigating the underlying molecular mechanisms. In *A. fumigatus*, the main targets of the azoles are Cyp51 proteins, encoded by two different, but related genes sharing 63% sequence identity, *cyp*51A and *cyp*51B [21]. The most frequent resistance mechanism is related to modifications in the azole target (Cyp51A, a 14α sterol demethylase), although other mechanisms within *A. fumigatus* have been investigated.

## 5. Cyp51A Mutations

Up to now, most of the *A. fumigatus* azole resistant strains have been associated with point mutations or overexpression of *cyp*51A. The *cyp*51A encodes a 14α-sterol-demethylase, a key enzyme in the ergosterol biosynthesis pathway [22]. Ergosterol is the main component of fungal cell membranes. Triazoles bind with one of the nitrogen atoms of the triazole ring to the iron atom in the heme group located at the active site of Cyp51A [22]. This way, demethylation of C-14 of lanosterol is blocked, and ergosterol is not synthesized. Lack of ergosterol alters membrane fluidity and leads to fungal cell death [1]. Several single-nucleotide polymorphisms (SNPs), responsible for *cyp*51A amino acid substitutions, with or without tandem repeats in the promoter region of the gene, have been described. Both mechanisms affect the binding of azoles to the enzyme and lead to the development of resistance. 

There are a few point mutations located at hot spot codons, whose link to azole resistance has been corroborated: (i) those associated with glycine 54 (G54), linked to cross-resistance to ITC and POS [23,24]; and (ii) amino acid substitutions at methionine 220 (M220), associated with different patterns of reduced susceptibility for triazoles [25]. Mutations in glycine 138 (G138), causing simultaneous resistance to itraconazole and voriconazole [26], and glycine 448 (G448S), resulting in VRC resistance, with some reduction in ITC and POS susceptibility, have also being reported in several studies [27,28,29]. Other point mutations, such as P216L, F219C, F219I, A284T, Y431C, G432S and G434C, have been occasionally described related to azole resistance, but further research is needed in order to confirm its role in the development of resistance [17,18,30,31,32,33,34,35,36]. In addition, a group of polymorphisms resulting in amino acid changes (F46Y, M172V, N248T, D255E and E427K) is frequently reported, alone or in combination, related to different patterns of susceptibility (they have been detected in azole susceptible and resistant strains), with consistently higher MICs than the wild type strains, although not always exceeding the breakpoint for resistance. More research is needed in order to determine the implication of each amino acid substitution (if any) in the azole profile shown by these strains (Table 1). All of these point mutations are generally described in strains isolated from patients that have been undergoing azole treatment.

A second group of *cyp*51A alterations with different resistance mechanisms has been reported, being normally described as panazole resistant. In *A. fumigatus*, this type of azole cross-resistance depends on specific mutations in *cyp*51A in combination with alterations in the promoter region, leading to multiazole-resistant strains [12,37,38]. These mechanisms are generated by combinations of *cyp*51A modifications: (i) the integration of a 34-bp tandem repeat (TR_34_) in the promoter region of the gene, leading to an overexpression of *cyp*51A along with a substitution of leucine 98 to histidine (TR_34_/L98H) [37]; this alteration is the most frequently identified resistance mechanism found in environmental *A. fumigatus* strains [39]; (ii) a 46-bp tandem repeat insertion in the promoter region and substitutions of tyrosine 121 to phenylalanine and threonine 289 to alanine (TR_46_/Y121F/T289A) [40], which is related to VRC resistance; and (iii) a 53-bp tandem repeat in the promoter region without any *cyp*51A amino acid substitution [41,42].

One of the first studies on azole cross-resistance in *A. fumigatus* was performed in 17 clinical *A. fumigatus* isolates that were ITC resistant. These strains showed cross-resistance between ITC and POS, which have a similar molecule structure, but not with VRC [43,44]. Cross-resistance between azoles was studied by Howard et al. showing that 74% of the ITC resistant isolates studied were cross-resistant to POS and 65% to VRC [17]. The newest triazole isavuconazole has shown higher MICs in strains with reduced susceptibilities to other triazoles and presented a high degree of correlation with VRC susceptibility results [45]. In addition, other azole fungicides are widely used for crop protection (DMIs), which exhibit a related molecule structure to medical triazoles, leading to development of cross-resistance with azole in clinical use [46].

## 6. Azole Resistance Mechanisms are *cyp*51A Independent

Although triazole resistance in *A. fumigatus* is mainly attributed to *cyp*51A target mutations, a recent survey of resistant isolates in Manchester showed that >50% of resistant isolates had no mutation in *cyp*51A or its promoter [98]. There is also a reported case of a Dutch patient with chronic granulomatous disease treated with azole-echinocandin combination therapy, whose resistant isolate revealed a four-to-five-fold increased expression of *cyp*51A without having any *cyp*51A alterations [2]. Therefore, other mechanisms of resistance in clinical azole-resistant isolates without *cyp*51A mutations need to be explored. 

Overexpression of *cyp51B*. In *A. fumigatus*, Cyp51 proteins are encoded by two different, but related genes sharing 63% sequence identity, *cyp*51A and *cyp*51B [21]. As described before, most of the azole-resistant strains have alterations in *cyp*51A; however, the role of *cyp*51B in *A. fumigatus* azole resistance remains unclear. Several *cyp*51B polymorphisms/mutations have been observed, but have never been linked to resistance. Only one study with a clinical azole-resistant isolate without *cyp*51A mutation or over-expression showed an over-expression of *cyp*51B [99]. Further studies are required to clearly define the relationship between this mechanism and azole resistance. 

Overexpression of efflux pumps. Fungi have to beat intracellular toxin accumulation in order to successfully colonize human hosts [1]. This is achieved by efflux pumps, of which there are two main categories: ATP-binding cassette (ABC) proteins, primary transporters that take advantage of ATP hydrolysis, and major facilitator superfamily (MFS) pumps, secondary transporters that use the proton-motive force across the plasma membrane [100]. In *A. fumigatus*, at least 49 ABC family transporters and 278 MFS genes have been described, which is more than four-times the number identified in yeasts like *Saccharomyces cerevisiae* [101]. However, in *A. fumigatus*, despite the great number of existing genes encoding transporters, little is known about the connection between ABC or MFS efflux pumps and triazole resistance. To date, only five transporter genes are known to be related to azole resistance: *AfuMDR1*, *AfuMDR2*, *AfuMDR3*, *AfuMDR4* and *AtrF*.

*AfuMDR1* and *AfuMDR2* ATP-binding cassette transporters were the first described, raising the possibility that these two genes could be directly involved in drug efflux in *A. fumigatus* [102]. Another ABC transporter, *atrF*, was cloned from a clinical isolate of *A. fumigatus* resistant to ITC, and five-fold higher levels of *atrF* mRNA compared to those in susceptible strains were revealed [103]. *AfuMDR3* and *AfuMDR4* were identified to be connected with triazole resistance in a study where resistant *A. fumigatus* mutants showed either constitutive high-level expression of both transporters or induction of expression when exposed to ITC. Two out of 23 mutants seemed to be ITC resistant due to overexpression of these genes, although evidence of a direct relationship between them and an ITC resistant phenotype is lacking. *AfuMDR3* has great similarity to MFS, and *AfuMDR4* is a member of the ABC proteins family [24]. Additionally, *AfuMDR4* has been shown to be induced with VRC in complex *A. fumigatus* biofilm populations and that this contributes to azole resistance [104]. Furthermore, exposure of a clinical azole-susceptible *A. fumigatus* isolate to VRC showed upregulation of five transporters of the ABC superfamily (*abcA*-*E*) and three of the MFS (*mfsA*-*C*) [105]. Lastly, a demonstrated link between transporters and azole resistance was the azole-induced expression of *cdr1B*. A *cdr*1B deleted mutant resulted in a four-fold susceptibility reduction in ITC MICs in an *A. fumigatus* clinical resistant isolate [106]. However, further studies are warranted in order to properly understand the relationship between the overexpression of pump efflux and azole resistance mechanisms in *A. fumigatus*.

Cholesterol import. The import of exogenous cholesterol under aerobic conditions, as a substitute for ergosterol after azole treatment, has also been described as a mechanism of resistance. The activity of ITC against *A. fumigatus* is compromised when cholesterol serum in RPMI medium is present [107]. In *A. fumigatus*, a sterol-regulatory element binding protein (*SrbA*) that plays a role in the azole resistance by *erg11* (*cyp5*1A) regulation has been characterized [108]. The *srb*A null mutant (∆*srb*A) was highly susceptible to FLC and VRC, which was explained by a reduction in *erg*11A transcript in response to both azoles. However, further studies on the genetic regulatory network mediated by *Srb*A in *A. fumigatus* and its role in triazole drug interactions need to be carried out [109,110]. 

Role of Hsp90. Heat shock protein 90 (Hsp90) is a eukaryotic molecular chaperone that helps crucial regulatory proteins in their folding, transport and maturation steps under environmental stress. Its involvement in the resistance of *Candida albicans* to azole and echinocandin antifungals is well established, but the function of Hsp90 in *A. fumigatus* remains unclear [111]. Using *S. cerevisiae* mutants expressing different levels of this chaperone, it was revealed that Hsp90 potentiates the acquisition of azole resistance and plays a key role in its continuance once it has been acquired. In *C. albicans* and *Aspergillus terreus*, Hsp90 inhibitors can beat azole and echinocandin resistance in vivo [112]. However, the mechanisms by which Hsp90 controls these functions remain to be fully investigated. 

HapE mutation. Another described mechanism is caused by a mutation in *HapE*, a CCAAT-binding transcription factor complex subunit. Two isogenic isolates with the wild-type *cyp*51A genotype, one azole susceptible isolated before treatment and the second with a resistant phenotype isolated post-treatment, were whole-genome sequenced in order to detect the resistance conferring mutation. Six out of a sixty-nine of identified point mutations in protein-coding regions were confirmed, and sexual crossing experiments revealed that a P88L substitution in HapE was the only one leading to resistance in progeny. This mutation in HapE can lead to a resistant phenotype by itself, as it was proven by cloning the mutated *hapE* gene into an azole-susceptible reference strain. This increase in resistance has been suggested to be due to a gain of function mutation if the mutated Hap-complex binds to a CCAAT-box in the promoter region of *cyp*51A and induces its expression [113].

## 7. Prevalence of Azole Resistance in *Aspergillus fumigatus* throughout the World

**Figure 1:**
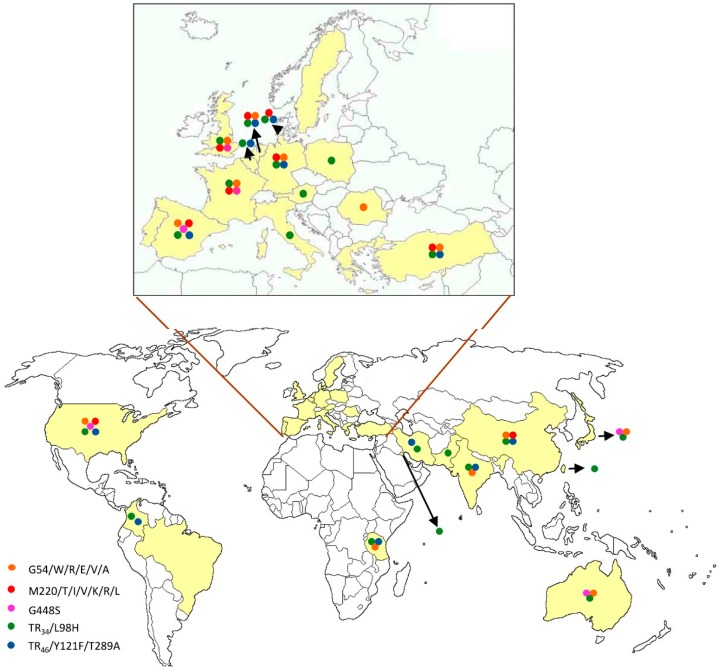
Worldwide distribution of azole resistance in *A. fumigatus* by mechanisms.

To date, Europe is the continent with the highest reported azole resistance in *A. fumigatus* (Table 2). Two reports in the late 2000s in the Netherlands and UK raised the alarm about an increase of azole resistance cases. The first one, in 2007, involved a series of Dutch patients suffering IA caused by panazole resistant strains, even those who had not been under azole treatment. One new resistance mechanism was found in these strains, TR_34_/L98H [37,38]. The second study, in 2009, described a wide range of *cyp*51A mutations found in patients in the U.K., becoming clear that a dramatic increase in azole resistance in *A. fumigatus* was occurring [17]. Since then, azole resistant cases in clinical samples have been reported in almost every European country, including Austria [70], Belgium [68,76,92,94], Denmark [35,61,66,70], France [19,27,30,48,50,73,91,114], Germany [32,47,51,60,72], Greece [115], Italy [36], The Netherlands [18,20,37,38,40,41,53,65,67,74,75,76], Poland [69,116], Portugal [117], Romania [118], Spain [12,23,25,29,34,37,49,63,70,93,119], Sweden [120], Turkey [71] and the UK [17,26,31,33,65]. Even though G54 and M220 point mutations have been occasionally reported in Europe since they were described [12,17,18,20,23,25,32,35,48,50,51,60,71], the TR_34_/L98H is by far the most common mutation found, both in environmental and clinical samples. Since its first report in 2007 in Spanish and Dutch isolates [37], TR_34_/L98H has been detected across Europe (Figure 1) [12,32,35,38,41,48,50,51,53,60,67,69,71,75]. In 2009 a new resistance mechanism, TR_46_/Y121F/T289A, was identified in The Netherlands [40]. Since then, it has also been reported in several countries [39,51,60,66,67,75,76,91,92,93]. Azole resistance in environmental strains in Europe has been commonly detected, with TR_34_/L98H and TR_46_/Y121F/T289A being the most often described mechanisms (Figure 1), and therefore, their emergence has been related with the extensive use of agricultural fungicides. Van der Linden et al. found that out of 140 environmental resistant strains, 14 had the TR_46_/Y121F/T289A mechanism, while 126 had TR_34_/L98H [40]. In Germany, an analysis of 455 environmental isolates revealed 45 that harbored the TR_34_/L98H mutation and six TR_46_/Y121F/T289A [47]. Another analysis reported 16% resistance (to ITC and POS) in environmental *A. fumigatus* isolates in Italy [36]. Other, less frequent point mutations have been described as related to the azole-resistant phenotype, but further research is needed in order to confirm it.

Reports from Asiatic countries suggest that triazole resistance rates in Asia are lower than in Europe (Table 2). The first two reports describing azole resistance in *A. fumigatus* in this area were published in 2005. One was from clinical strains from Taiwan, where two out of 40 isolates showed azole resistance, but mutations in *cyp*51A were not investigated [125]; and the second one was based on six isogenic isolates obtained from a Chinese patient treated with azoles and suffering from lung aspergilloma. ITC resistance was found in four post-treatment isolates, one of them with a M220I mutation and the rest with G54R [54]. Several other cases have been reported since then. The ARTEMIS global antifungal susceptibility program included more than 100 medical centers worldwide and detected several clinical isolates from China that had a TR_34_/L98H resistance mechanism [123]. This alteration has also been reported in 7.9% of the multi-azole resistant strains isolated from azole-naïve patients in Taiwan [87] and in three out of fourteen resistant clinical isolates in Pakistan [85]. In contrast, TR_34_/L98H has not been described in Japan, with reports showing a low azole resistant strains rate. Kikuchi et al. found three resistant isolates out of 171 *A. fumigatus* clinical strains isolated between 1987 and 2008 [121]. Some novel mutations have been reported in this country, such as the P216L [97] or F332K [126], and the G448S and TR_46_/Y121F/T289A mechanisms were recently identified in Japan for the first time [64,95]. Azole resistance prevalence in *A. fumigatus* is also low in India, where three studies revealed the presence of TR_34_/L98H as the resistance mechanism in clinical isolates: 44 out of 630 (6.9%), two out of 103 (1.9%) and 10 out of 685 (1.5%) [3,80,81]. Similar findings have been observed in Middle East countries, like Iran (3.5% of clinical samples) [84] or Kuwait (two out of 16 clinical isolates and one out of 50 environmental isolates) [77]. Azole resistance in environmental strains in Asia is also lower than in Europe (Table 2). In fact, a recent report on the use of azole fungicides on a pumpkin farm revealed no azole resistance in 50 *A. fumigatus* isolates [127]. Several environmental studies have been performed in India, describing the TR_46_/Y121F/T289A mechanism for the first time in Asia in isolates from agricultural fields [82] and showing that 44 out of 630 *A. fumigatus* sampled from the soil of paddy fields, tea gardens, cotton trees, flower pots and indoor air of hospitals were resistant and harbored the TR_34_/L98H resistance mechanism [81]. A report from Iran described 12.2% of environmental resistant strains [79], and in Kuwait, 7% of environmental samples were also resistant [78], all of them carrying TR_34_/L98H. This difference in environmental azole resistance rates between Asia and Europe could be due to the lower use of azole fungicides in Asian countries [128].

The first study involving a large number of isolates in the U.S. included 181 *A. fumigatus* isolates from transplant patients with proven IA from 2001–2006 (multicenter prospective study). Only one of these isolates was triazole resistant [122] and indicates a low azole resistance prevalence in this country. Similarly, 1096 *A. fumigatus* clinical strains from all over the U.S. collected between 2011 and 2013 were studied; 51 of them were sequenced for *cyp*51A mutations. One isolate possessed the M220I mutation in *cyp*51A, and 13 isolates had another mutation, I242V; TR_34_/L98H was not identified [62]. A recent comprehensive study in the U.S. included 220 clinical *A. fumigatus* isolates obtained from 2001–2014, with the description of two isolates harboring TR_34_/L89H mutations and the other two with TR_46_/Y121F/T289A. This was the first report of both resistance mechanisms in *A. fumigatus* isolates in the United States. Other point mutations detected in the 26 azole resistant strains were G54R/W/E, M220I/K/V, G138S/C, G448S and F219S [58]. To our knowledge, no environmental sample studies have been reported in this country yet, but there is also lower use of fungicides in the U.S. as compared to Europe [128]. 

Some investigations have been carried out in South American countries, such as Brazil, where six out of 170 clinical *A. fumigatus* collected between 2000 and 2012 showed azole resistance, but neither the TR_34_/L98H nor the TR_46_/Y121F/T289A mechanisms were found [129]. An environmental study has been carried out in Colombia, known to be the fourth country in the world for pesticide use, 30% of which are fungicides. Sixty soil samples from flower beds and flower fields were analyzed, describing one TR_34_/L98H, 17 TR_46_/Y121F/T289A and one TR53 isolates [88]. Colombia is the second biggest flower exporter after The Netherlands, which could explain the high environmental azole resistance rate in *A. fumigatus* [129]. 

Azole resistance has also been reported in Africa; 15 out of 108 environmental samples taken in Tanzania were azole resistant, 11 of them with the TR_34_/L98H mutation and four with TR_46_/Y121F/T289A [90]. Another study in the same country describes G54E as responsible for 46.4% of resistant environmental *A. fumigatus* isolates from this country [118]. To our knowledge, no reports from clinical samples have been published in Africa yet. 

In Australia, 418 *A. fumigatus* clinical strains were collected from 2000–2013, revealing nine isolates with reduced susceptibility to ITC, VRC and POS. All of them had between two and five amino acid substitutions, including G54R, F46Y, Y431S, G448S, M172V, N248T, D255E, E427K and TR_34_/L98H, the latter being identified in two isolates. The first TR_34_/L98H *A. fumigatus* was recovered in 2004, and it is believed to be Australian-acquired in a patient on long-term ITC therapy, while the second isolate was suspected to have been acquired in Europe while the patient was travelling in 2012 [59]. 

## 8. Azole Resistance in Other *Aspergillus* Species

A shift in epidemiology of fungal infections towards a greater number of species able to cause disease in humans has occurred [130]. The leading cause of IA is *A. fumigatus* (85%), followed by *A. flavus* (5%–10%), *A. terreus* (2%–10%) and *A. niger* (2%–3%) [100]. However, the use of molecular tools has led to the description of new species within the genus *Aspergillus*. Some of these species are considered cryptic or sibling because they are difficult to differentiate by classical methods, and they have been frequently misidentified. Their prevalence in the clinical setting has been reported to be between 10% and 15% in two studies. The TRANSNET (Transplant-Associated Infection Surveillance Network) study included 218 *Aspergillus* isolates from transplant recipients with proven or probable IA from 2001–2006 from the U.S. and documented an 11% cryptic species [131]. The FILPOP study (population-based survey of filamentous fungi) from Spain described 15% cryptic species among 323 isolates analyzed [119]. The importance of these cryptic species in the clinical setting is based on their different susceptibility profile, as they are frequently more resistant to the antifungals available [132]. As these cryptic species are difficult to differentiate, it has been recommended that when using classical identification methods in the clinical setting, an *Aspergillus* isolate should be classified to the “species complex” level, thereby accounting for gathering all closely-related cryptic species.

The *Aspergillus fumigatus* complex includes several species that have been reported in human infections: *Aspergillus lentulus*, *A. udagawae* (syn. *Neosartorya udagawae*), *A. pseudofischeri* (syn. *Neosartorya pseudofischeri*), *A. viridinutans*, *A. fumigatiaffinis*, *A. fumisynnematus* and *A. hiratsukae* (syn. *Neosartorya hiratsukae*) [119,131,133]. Antifungal susceptibility testing of these species revealed heterogeneous patterns. *Aspergillus lentulus*, *A. fumigatiaffinis* and *A. udagawae* show high MICs for AmB, with the first two of these also having high MICs for azoles, but *A. udagawae* has intermediate values for VRC and low MICs for ITC or PCZ. *Aspergillus viridinutans* and *A. pseudofischeri* have reduced susceptibility for azoles, but not for AmB, and *A. hiratsukae* and *A. fumisynnematus* are susceptible to all drugs [132,133,134,135,136,137]. 

The *A. niger* includes *A. tubingensis*, the second most frequent species of the complex in clinical isolates, and has been found with similar prevalence as *A. niger* in some studies [76,119]. *Aspergillus awamori* and *A. foetidus* have also been described in clinical samples, although there is debate about their classification as new species or subspecies of *A. niger* [138]. The susceptibility profile of these species is isolate dependent, and three patterns have been described regarding ITC: low MICs, high MICs and isolates that show a paradoxical effect (which are able to grow in the presence of high antifungal concentrations, but remain fully susceptible at intermediate-to-low concentrations [139]) for this antifungal [140]. *Aspergillus niger* and *A. awamori* have been reported to have higher MICs to azoles than *A. tubingensis* [141]. 

*Aspergillus flavus* is the second most common *Aspergillus* causing IA, and it is reported as the most prevalent in countries with arid climates, such as those in the Middle East, Africa and Southeast Asia, as it is capable of surviving in extreme conditions [142]. *Aspergillus alliaceus* is part of the *A. flavus* complex. This species has elevated MICs to AmB and echinocandins, but is variable regarding azoles. The first report describing *A. alliaceus* stated that ITC was the most active antifungal in vitro against this mold [143], but the first study reporting IA caused by *A. alliaceus* (together with *A. flavus*) defined VRC as the best option for treatment, as the isolate tested was resistant to ITC and POS [144]. VRC resistance has also been reported in clinical strains of *A. flavus*, and T788G and Y319H mutations in the *cyp*51C gene have been found to be associated with these high MICs to VRC [145,146].

*Aspergillus terreus* shows high MICs to AmB both in vitro [147,148] and in vivo [149], and reduced susceptibility to azoles has also been described. A study from 2012 reports a *cyp*51A mutation, M217I, in some clinical *A. terreus* isogenic isolates causing ITC resistance [150]. The *A. terreus* complex includes *Aspergillus alabamensis*, *A. floccosus*, *A. neoafricanus*, *A. aureoterreus A. hortai*, *A. pseudoterreus* [151] and *Aspergillus citrinoterreus*. They all have high MICs to AmB, but *A. hortai* and *A. citrinoterreus* are more susceptible to azoles than *A. terreus* [152,153]. 

The *Aspergillus ustus* complex is known for its elevated MICs to most drugs. *Aspergillus calidoustus* was described in 2008 as being able to grow at 37 °C, in contrast to *A. ustus*, and has been isolated from human infections [154]. Triazoles have been reported to be inactive in vitro against *A. calidoustus* [122], and the same has been reported of other antifungal classes, so it is considered a multiresistant species. Other cryptic species with high MICs to all antifungals in this complex are *A. keveii* and *A. insuetus*, also isolated from clinical samples [155].

## 9. Treatment Options

Mortality rates in patients infected with azole-resistant strains (ITC > 2 µg/mL, VRC > 2 µg/mL, POS > 0.5 µg/mL, determined by the CLSI reference method) are higher than those affected with azole-susceptible ones (88% vs. 30%–50%) [53]. As mentioned above, VRC is the primary treatment for IA, but liposomal amphotericin B (l-AMB) is recommended as an alternative therapy [156]. l-AMB was demonstrated to develop no cross-resistance in a murine model of disseminated azole-resistant aspergillosis, being either active against azole-susceptible or azole-resistant strains [157]. However, this drug is not recommended to treat infections caused by *A. terreus* or other AMB-resistant species. Another approach to consider is an antifungal combination therapy that leads to a synergistic response. A great number of in vitro, in vivo and clinical studies have tested various antifungal combinations and found some of them effective against *A. fumigatus* [158]. Recent studies have focused on the combination of an azole, normally VRC, with an echinocandin, both for azole-susceptible and azole-resistant *A. fumigatus* strains. The efficacy of this combined therapy mainly relies on anidulafungin (AND) [159], which is currently not licensed for the treatment of IA. In one clinical study, mortality rates were 27.5% for monotherapy and 19.3% for combined therapy of VRC and AND [160]. In a murine model, AND was successful against 45% of VRC-resistant strains when used as monotherapy [161]. Further studies for combined therapy are warranted in order to find alternative treatment options, given the limitations of current monotherapy. Although azole-resistant strains have been present in clinical samples for more than two decades, it has been suggested that first-line therapy should remain as azoles whilst local azole resistance prevalence remains below 10% [162]. Still, therapeutic options for IA should be revised taking this issue into account. 

## 10. Conclusions and Recommendations for Clinical Practice

Clinical and environmental triazole resistance in *Aspergillus* species is a growing public health concern that has become a worldwide problem. Even though the highest rates of triazole resistance have been described in Europe, several cases have been reported in every continent, and new resistance mechanisms are being described. Despite *A. fumigatus* being the most common *Aspergillus* species, triazole resistance has also been identified in many cryptic species of *Aspergillus*. Therefore, the morphological identification of an isolate cannot always drive the treatment strategy. We recommend performing antifungal susceptibility testing on every *Aspergillus* isolate associated with IA in order to select the best antifungal treatment. In addition, the prevalence of resistant strains should be investigated in every country to understand the prevalence of resistance and to adjust therapeutic options where high rates of resistant isolates are present. Moreover, the development of molecular methods to detect azole resistance in culture-negative infections could be very useful in laboratory practice. 

It is important to investigate more extensively the origin of environmental samples that are resistant to triazoles, since measures to reduce the use of agricultural azoles could be an important step in reducing resistance rates in the clinical setting, as stated in the technical report published by the European Centre for Disease prevention and Control (ECDC) [163].

## Figures and Tables

**Table 1:** Described *Aspergillus fumigatus cyp*51A mutations.

*cyp*51A Amino Acid No./Change	Continents	References
Described in resistant strains with a known mechanism		
G54/W/R/E/V/A	Europe	[12,17,18,23,32,47,48,49,50,51,52,53]
	Asia	[3,54,55,56,57]
	America	[58]
	Oceania	[59]
M220/T/V/I/K/R/L	Europe	[12,17,20,25,32,33,35,47,48,50,52,60,61]
	Asia	[54,57]
	America	[58,62]
G448S	Europe	[17,27,29,63]
	Asia	[64]
	America	[58]
	Oceania	[59]
Promoter tandem insertion + *cyp*51A amino acid No./change		
TR_34_/L98H with or without S297T/F497I	Europe	[12,17,20,32,35,36,37,38,40,47,48,50,51,52,53,60,65,66,67,68,69,70,71,72,73,74,75,76]
	Asia	[3,77,78,79,80,81,82,83,84,85,86,87]
	America	[58,88,89]
	Africa	[90]
	Oceania	[59]
TR_46_/Y121F/T289A with or without S297T/F497I	Europe	[40,47,51,52,60,66,67,75,76,91,92,93,94]
	Asia	[82,95,96]
	America	[58,88,89]
	Africa	[90]
TR53	Europe	[41]
	America	[88]
Described in resistant strains with an unknown mechanism		
G138/C/S	Europe	[17,26,31]
	America	[58]
Described both in resistant and susceptible strains		
F46Y/M172V/N248T/D255E/E42 7K or some other combinations	Europe	[17,33,34,36,53,61,65,71]
	Asia	[3]
	Oceania	[59]
F46Y/M172V/E427K	Europe	[12,17,33,34,74,75]
Occasionally described in susceptible or resistant strains		
P216L	[17,18,53,61,75,97]	
F219/S/C/I	[18,32,53,58]	
I242V	[12,62]	
N248K	[12,34,83]	
Y431/S/C	[17,31,35,59]	
G432/S/A	[30,83]	
G434C	[17,31]	

**Table 2:** Azole resistance prevalence in *A. fumigatus* by continent and/or country. Only significant publications with more than 50 isolates tested are reported.

Continent/Country	% Resistance	Source of the Isolates	References
Europe			
Belgium	5.7	C	[76]
France	0.85–10.6	C	[30,48,50]
Germany	1.1–12	C and E	[32,47,60]
Netherlands	2.1–20	C and E	[20,53,67,74]
Poland	2.25	C	[69]
Spain	1.8	C	[63]
Turkey	10.2	C	[71]
United Kingdom	6.6–28	C	[17,33]
Other continents			
Asia *	1.9–11.1	C and E	[55,77,78,80,81,82,83,84,85,86,121]
Africa (Tanzania)	13.9	E	[90]
America (USA)	0.6–11.8	C	[58,122]
Oceania (Australia)	2.6	C	[59]
International surveillance studies			
America-Asia-Australia-Europe	1.4–5.8	C and E	[52,70,123,124]

C = clinical strains, E = environmental strains; * including China, India, Iran, Japan, Kuwait and Pakistan.

## References

[1] Chowdhary A., Sharma C., Hagen F., Meis J.F. (2014). Exploring azole antifungal drug resistance in *Aspergillus fumigatus* with special reference to resistance mechanisms. Future. Microbiol..

[2] Arendrup M.C., Mavridou E., Mortensen K.L., Snelders E., Frimodt-Moller N., Khan H., Melchers W.J., Verweij P.E. (2010). Development of azole resistance in *Aspergillus fumigatus* during azole therapy associated with change in virulence. PLoS ONE.

[3] Chowdhary A., Sharma C., Kathuria S., Hagen F., Meis J.F. (2015). Prevalence and mechanism of triazole resistance in *Aspergillus fumigatus* in a referral chest hospital in Delhi, India and an update of the situation in Asia. Front. Microbiol..

[4] Kwon-Chung K.J., Sugui J.A. (2013). *Aspergillus fumigatus*—what makes the species a ubiquitous human fungal pathogen?. PLoS Pathog..

[5] Miceli M.H., Kauffman C.A. (2015). Isavuconazole: A new broad-spectrum triazole antifungal agent. Clin. Infect. Dis..

[6] Verweij P.E., Chowdhary A., Melchers W.J., Meis J.F. (2016). Azole resistance in *Aspergillus fumigatus*: Can we retain the clinical use of mold-active antifungal azoles?. Clin. Infect. Dis..

[7] Maschmeyer G., Haas A., Cornely O.A. (2007). Invasive aspergillosis: Epidemiology, diagnosis and management in immunocompromised patients. Drugs.

[8] Walsh T.J., Anaissie E.J., Denning D.W., Herbrecht R., Kontoyiannis D.P., Marr K.A., Morrison V.A., Segal B.H., Steinbach W.J., Stevens D.A. (2008). Treatment of aspergillosis: Clinical practice guidelines of the infectious diseases society of America. Clin. Infect. Dis..

[9] Howard S.J., Pasqualotto A.C., Denning D.W. (2010). Azole resistance in allergic bronchopulmonary aspergillosis and *Aspergillus* bronchitis. Clin. Microbiol. Infect..

[10] Cornely O.A., Maertens J., Winston D.J., Perfect J., Ullmann A.J., Walsh T.J., Helfgott D., Holowiecki J., Stockelberg D., Goh Y.T. (2007). Posaconazole vs. Fluconazole or itraconazole prophylaxis in patients with neutropenia. N. Engl. J. Med..

[11] Espinel-Ingroff A., Diekema D.J., Fothergill A., Johnson E., Pelaez T., Pfaller M.A., Rinaldi M.G., Canton E., Turnidge J. (2010). Wild-type MIC distributions and epidemiological cutoff values for the triazoles and six *Aspergillus* spp. For the CLSI broth microdilution method (M38-A2 document). J. Clin. Microbiol..

[12] Rodriguez-Tudela J.L., Alcazar-Fuoli L., Mellado E., Alastruey-Izquierdo A., Monzon A., Cuenca-Estrella M. (2008). Epidemiological cutoffs and cross-resistance to azole drugs in *Aspergillus fumigatus*. Antimicrob. Agents Chemother..

[13] EUCAST European committee on antimicrobial susceptibility testing Antifungal breakpoint tables for interpretation of MICs v 8.0. http://www.eucast.org/fileadmin/src/media/PDFs/EUCAST_files/AFST/Clinical_breakpoints/Antifungal_breakpoints_v_8.0_November_2015.pdf.

[14] Denning D.W., Venkateswarlu K., Oakley K.L., Anderson M.J., Manning N.J., Stevens D.A., Warnock D.W., Kelly S.L. (1997). Itraconazole resistance in *Aspergillus fumigatus*. Antimicrob. Agents Chemother..

[15] Chowdhary A., Kathuria S., Xu J., Meis J.F. (2013). Emergence of azole-resistant *Aspergillus fumigatus* strains due to agricultural azole use creates an increasing threat to human health. PLoS Pathog..

[16] Verweij P.E., Snelders E., Kema G.H., Mellado E., Melchers W.J. (2009). Azole resistance in *Aspergillus fumigatus*: A side-effect of environmental fungicide use?. Lancet Infect. Dis..

[17] Howard S.J., Cerar D., Anderson M.J., Albarrag A., Fisher M.C., Pasqualotto A.C., Laverdiere M., Arendrup M.C., Perlin D.S., Denning D.W. (2009). Frequency and evolution of azole resistance in *Aspergillus fumigatus* associated with treatment failure. Emerg. Infect. Dis..

[18] Camps S.M., van der Linden J.W., Li Y., Kuijper E.J., van Dissel J.T., Verweij P.E., Melchers W.J. (2012). Rapid induction of multiple resistance mechanisms in *Aspergillus fumigatus* during azole therapy: A case study and review of the literature. Antimicrob. Agents Chemother..

[19] Dannaoui E., Borel E., Monier M.F., Piens M.A., Picot S., Persat F. (2001). Acquired itraconazole resistance in *Aspergillus fumigatus*. J. Antimicrob. Chemother..

[20] Snelders E., van der Lee H.A., Kuijpers J., Rijs A.J., Varga J., Samson R.A., Mellado E., Donders A.R., Melchers W.J., Verweij P.E. (2008). Emergence of azole resistance in *Aspergillus fumigatus* and spread of a single resistance mechanism. PLoS Med..

[21] Mellado E., Diaz-Guerra T.M., Cuenca-Estrella M., Rodriguez-Tudela J.L. (2001). Identification of two different 14-alpha sterol demethylase-related genes (*cyp*51A and *cyp*51B) in *Aspergillus fumigatus* and other *Aspergillus* species. J. Clin. Microbiol..

[22] Snelders E., Karawajczyk A., Schaftenaar G., Verweij P.E., Melchers W.J. (2010). Azole resistance profile of amino acid changes in *Aspergillus fumigatus cyp*51A based on protein homology modeling. Antimicrob. Agents Chemother..

[23] Diaz-Guerra T.M., Mellado E., Cuenca-Estrella M., Rodriguez-Tudela J.L. (2003). A point mutation in the 14alpha-sterol demethylase gene *cyp*51A contributes to itraconazole resistance in *Aspergillus fumigatus*. Antimicrob. Agents Chemother..

[24] Nascimento A.M., Goldman G.H., Park S., Marras S.A., Delmas G., Oza U., Lolans K., Dudley M.N., Mann P.A., Perlin D.S. (2003). Multiple resistance mechanisms among *Aspergillus fumigatus* mutants with high-level resistance to itraconazole. Antimicrob. Agents Chemother..

[25] Mellado E., Garcia-Effron G., Alcazar-Fuoli L., Cuenca-Estrella M., Rodriguez-Tudela J.L. (2004). Substitutions at methionine 220 in the 14alpha-sterol demethylase (*cyp*51A) of *Aspergillus fumigatus* are responsible for resistance in vitro to azole antifungal drugs. Antimicrob. Agents Chemother..

[26] Howard S.J., Webster I., Moore C.B., Gardiner R.E., Park S., Perlin D.S., Denning D.W. (2006). Multi-azole resistance in *Aspergillus fumigatus*. Int. J. Antimicrob. Agents.

[27] Bellete B., Raberin H., Morel J., Flori P., Hafid J., Manhsung R.T. (2010). Acquired resistance to voriconazole and itraconazole in a patient with pulmonary aspergilloma. Med. Mycol..

[28] Manavathu E., Espinel-Ingroff A., Alangaden G., Chandrasekar P. Molecular studies on voriconazole resistance in a clinical isolate of *Aspergillus fumigatus*. Proceedings of 43rd Annual Interscience Conference on Antimicrobial Agents and Chemotherapy.

[29] Pelaez T., Gijon P., Bunsow E., Bouza E., Sanchez-Yebra W., Valerio M., Gama B., Cuenca-Estrella M., Mellado E. (2012). Resistance to voriconazole due to a G448S substitution in *Aspergillus fumigatus* in a patient with cerebral aspergillosis. J. Clin. Microbiol..

[30] Alanio A., Sitterle E., Liance M., Farrugia C., Foulet F., Botterel F., Hicheri Y., Cordonnier C., Costa J.M., Bretagne S. (2011). Low prevalence of resistance to azoles in *Aspergillus fumigatus* in a French cohort of patients treated for haematological malignancies. J. Antimicrob. Chemother..

[31] Albarrag A.M., Anderson M.J., Howard S.J., Robson G.D., Warn P.A., Sanglard D., Denning D.W. (2011). Interrogation of related clinical pan-azole-resistant *Aspergillus fumigatus* strains: G138C, Y431C, and G434C single nucleotide polymorphisms in *cyp*51A, upregulation of *cyp*51A, and integration and activation of transposon *Atf1* in the *cyp*51a promoter. Antimicrob. Agents Chemother..

[32] Bader O., Weig M., Reichard U., Lugert R., Kuhns M., Christner M., Held J., Peter S., Schumacher U., Buchheidt D. (2013). *cyp*51A-based mechanisms of *Aspergillus fumigatus* azole drug resistance present in clinical samples from Germany. Antimicrob. Agents Chemother..

[33] Bueid A., Howard S.J., Moore C.B., Richardson M.D., Harrison E., Bowyer P., Denning D.W. (2010). Azole antifungal resistance in *Aspergillus fumigatus*: 2008 and 2009. J. Antimicrob. Chemother..

[34] Escribano P., Recio S., Pelaez T., Bouza E., Guinea J. (2011). *Aspergillus fumigatus* strains with mutations in the *cyp*51A gene do not always show phenotypic resistance to itraconazole, voriconazole, or posaconazole. Antimicrob. Agents Chemother..

[35] Mortensen K.L., Jensen R.H., Johansen H.K., Skov M., Pressler T., Howard S.J., Leatherbarrow H., Mellado E., Arendrup M.C. (2011). *Aspergillus* species and other molds in respiratory samples from patients with cystic fibrosis: A laboratory-based study with focus on *Aspergillus fumigatus* azole resistance. J. Clin. Microbiol..

[36] Prigitano A., Venier V., Cogliati M., De L.G., Esposto M.C., Tortorano A.M. (2014). Azole-resistant *Aspergillus fumigatus* in the environment of Northern Italy, May 2011 to June 2012. Euro. Surveill.

[37] Mellado E., Garcia-Effron G., Alcazar-Fuoli L., Melchers W.J., Verweij P.E., Cuenca-Estrella M., Rodriguez-Tudela J.L. (2007). A new *Aspergillus fumigatus* resistance mechanism conferring in vitro cross-resistance to azole antifungals involves a combination of *cyp*51A alterations. Antimicrob. Agents Chemother..

[38] Verweij P.E., Mellado E., Melchers W.J. (2007). Multiple-triazole-resistant aspergillosis. N. Engl. J. Med..

[39] Vermeulen E., Lagrou K., Verweij P.E. (2013). Azole resistance in *Aspergillus fumigatus*: A growing public health concern. Curr. Opin. Infect. Dis..

[40] van der Linden J.W., Camps S.M., Kampinga G.A., Arends J.P., Debets-Ossenkopp Y.J., Haas P.J., Rijnders B.J., Kuijper E.J., van Tiel F.H., Varga J. (2013). Aspergillosis due to voriconazole highly resistant *Aspergillus fumigatus* and recovery of genetically related resistant isolates from domiciles. Clin. Infect. Dis..

[41] Hodiamont C.J., Dolman K.M., Ten Berge I.J., Melchers W.J., Verweij P.E., Pajkrt D. (2009). Multiple-azole-resistant *Aspergillus fumigatus* osteomyelitis in a patient with chronic granulomatous disease successfully treated with long-term oral posaconazole and surgery. Med. Mycol..

[42] Mellado E., Alcazar-Fuoli L., Pajkrt D., Verweij P.E., Melchers W.J., Cuenca-Estrella M., Rodriguez-Tudela J.L. Alterations of the *cyp*51A gene promoter contribute to *Aspergillus fumigatus* multiple triazole resistance. Proceedings of 47th Annual Interscience Conference on Antimicrobial Agents and Chemotherapy.

[43] Mosquera J., Denning D.W. (2002). Azole cross-resistance in *Aspergillus fumigatus*. Antimicrob. Agents Chemother..

[44] Xiao L., Madison V., Chau A.S., Loebenberg D., Palermo R.E., McNicholas P.M. (2004). Three-dimensional models of wild-type and mutated forms of cytochrome P450 14α-sterol demethylases from *Aspergillus fumigatus* and *Candida albicans* provide insights into posaconazole binding. Antimicrob. Agents Chemother..

[45] Gregson L., Goodwin J., Johnson A., McEntee L., Moore C.B., Richardson M., Hope W.W., Howard S.J. (2013). In vitro susceptibility of *Aspergillus fumigatus* to isavuconazole: Correlation with itraconazole, voriconazole, and posaconazole. Antimicrob. Agents Chemother..

[46] Snelders E., Camps S.M., Karawajczyk A., Schaftenaar G., Kema G.H., van der Lee H.A., Klaassen C.H., Melchers W.J., Verweij P.E. (2012). Triazole fungicides can induce cross-resistance to medical triazoles in *Aspergillus fumigatus*. PLoS ONE.

[47] Bader O., Tunnermann J., Dudakova A., Tangwattanachuleeporn M., Weig M., Gross U. (2015). Environmental isolates of azole-resistant *Aspergillus fumigatus* in Germany. Antimicrob. Agents Chemother..

[48] Burgel P.R., Baixench M.T., Amsellem M., Audureau E., Chapron J., Kanaan R., Honore I., Dupouy-Camet J., Dusser D., Klaassen C.H. (2012). High prevalence of azole-resistant *Aspergillus fumigatus* in adults with cystic fibrosis exposed to itraconazole. Antimicrob. Agents Chemother..

[49] Escribano P., Recio S., Pelaez T., Gonzalez-Rivera M., Bouza E., Guinea J. (2012). In vitro acquisition of secondary azole resistance in *Aspergillus fumigatus* isolates after prolonged exposure to itraconazole: Presence of heteroresistant populations. Antimicrob. Agents Chemother..

[50] Morio F., Aubin G.G., Danner-Boucher I., Haloun A., Sacchetto E., Garcia-Hermoso D., Bretagne S., Miegeville M., Le P.P. (2012). High prevalence of triazole resistance in *Aspergillus fumigatus*, especially mediated by TR34/L98H, in a French cohort of patients with cystic fibrosis. J. Antimicrob. Chemother..

[51] Steinmann J., Hamprecht A., Vehreschild M.J., Cornely O.A., Buchheidt D., Spiess B., Koldehoff M., Buer J., Meis J.F., Rath P.M. (2015). Emergence of azole-resistant invasive aspergillosis in HSCT recipients in Germany. J. Antimicrob. Chemother..

[52] van der Linden J.W., Arendrup M.C., Warris A., Lagrou K., Pelloux H., Hauser P.M., Chryssanthou E., Mellado E., Kidd S.E., Tortorano A.M. (2015). Prospective multicenter international surveillance of azole resistance in *Aspergillus fumigatus*. Emerg. Infect. Dis..

[53] van der Linden J.W., Snelders E., Kampinga G.A., Rijnders B.J., Mattsson E., Debets-Ossenkopp Y.J., Kuijper E.J., van Tiel F.H., Melchers W.J., Verweij P.E. (2011). Clinical implications of azole resistance in *Aspergillus fumigatus*, The Netherlands, 2007–2009. Emerg. Infect. Dis..

[54] Chen J., Li H., Li R., Bu D., Wan Z. (2005). Mutations in the *cyp5*1A gene and susceptibility to itraconazole in *Aspergillus fumigatus* serially isolated from a patient with lung aspergilloma. J. Antimicrob. Chemother..

[55] Tashiro M., Izumikawa K., Hirano K., Ide S., Mihara T., Hosogaya N., Takazono T., Morinaga Y., Nakamura S., Kurihara S. (2012). Correlation between triazole treatment history and susceptibility in clinically isolated *Aspergillus fumigatus*. Antimicrob. Agents Chemother..

[56] Tashiro M., Izumikawa K., Minematsu A., Hirano K., Iwanaga N., Ide S., Mihara T., Hosogaya N., Takazono T., Morinaga Y. (2012). Antifungal susceptibilities of *Aspergillus fumigatus* clinical isolates obtained in Nagasaki, Japan. Antimicrob. Agents Chemother..

[57] Xu H., Chen W., Li L., Wan Z., Li R., Liu W. (2010). Clinical itraconazole-resistant strains of *Aspergillus fumigatus*, isolated serially from a lung aspergilloma patient with pulmonary tuberculosis, can be detected with real-time PCR method. Mycopathologia.

[58] Wiederhold N.P., Gil V.G., Gutierrez F., Lindner J.R., Albataineh M.T., McCarthy D.I., Sanders C., Fan H., Fothergill A.W., Sutton D.A. (2016). First detection of TR34/L98H and TR46/Y121F/T289A *cyp*51 mutations in *Aspergillus fumigatus* isolates in the United States. J. Clin. Microbiol..

[59] Kidd S.E., Goeman E., Meis J.F., Slavin M.A., Verweij P.E. (2015). Multi-triazole-resistant *Aspergillus fumigatus* infections in Australia. Mycoses.

[60] Fischer J., van Koningsbruggen-Rietschel S., Rietschel E., Vehreschild M.J., Wisplinghoff H., Kronke M., Hamprecht A. (2014). Prevalence and molecular characterization of azole resistance in *Aspergillus* spp. Isolates from German cystic fibrosis patients. J. Antimicrob. Chemother..

[61] Zhao Y., Stensvold C.R., Perlin D.S., Arendrup M.C. (2013). Azole resistance in *Aspergillus fumigatus* from bronchoalveolar lavage fluid samples of patients with chronic diseases. J. Antimicrob. Chemother..

[62] Pham C.D., Reiss E., Hagen F., Meis J.F., Lockhart S.R. (2014). Passive surveillance for azole-resistant *Aspergillus fumigatus*, United States, 2011–2013. Emerg. Infect. Dis..

[63] Escribano P., Pelaez T., Munoz P., Bouza E., Guinea J. (2013). Is azole resistance in *Aspergillus fumigatus* a problem in Spain?. Antimicrob. Agents Chemother..

[64] Toyotome T., Fujiwara T., Kida H., Matsumoto M., Wada T., Komatsu R. Susceptibility to azoles in clinical isolates of *Aspergillus fumigatus* and *A. tubingensis* fron Obihiro, Japan. Proceedings of 7th Advances Against Aspergillosis.

[65] Abdolrasouli A., Rhodes J., Beale M.A., Hagen F., Rogers T.R., Chowdhary A., Meis J.F., Armstrong-James D., Fisher M.C. (2015). Genomic context of azole resistance mutations in *Aspergillus fumigatus* determined using whole-genome sequencing. MBio..

[66] Astvad K.M., Jensen R.H., Hassan T.M., Mathiasen E.G., Thomsen G.M., Pedersen U.G., Christensen M., Hilberg O., Arendrup M.C. (2014). First detection of TR46/Y121F/T289A and TR34/L98H alterations in *Aspergillus fumigatus* isolates from azole-naive patients in Denmark despite negative findings in the environment. Antimicrob. Agents Chemother..

[67] Fuhren J., Voskuil W.S., Boel C.H., Haas P.J., Hagen F., Meis J.F., Kusters J.G. (2015). High prevalence of azole resistance in *Aspergillus fumigatus* isolates from high-risk patients. J. Antimicrob. Chemother..

[68] Jeurissen A., Cooreman S., Van K.W., Van L.J., Vanhove P., Lagrou K., Heytens L. (2012). Invasive pulmonary aspergillosis due to a multi-azole resistant *Aspergillus fumigatus*. Acta Clin. Belg..

[69] Kurzyk E.M., Nawrot U., Mroczynska M., Wlodarczyk K., Ussowicz M., Zdziarski P., Arendrup M.C., Brillowska-Dabrowska A. Detection of clinical *Aspergillus fumigatus* isolates resistant to triazoles. Proceedings of the 7th Trends in Medical Mycology.

[70] Mortensen K.L., Mellado E., Lass-Florl C., Rodriguez-Tudela J.L., Johansen H.K., Arendrup M.C. (2010). Environmental study of azole-resistant *Aspergillus fumigatus* and other aspergilli in Austria, Denmark, and Spain. Antimicrob. Agents Chemother..

[71] Ozmerdiven G.E., Ak S., Ener B., Agca H., Cilo B.D., Tunca B., Akalin H. (2015). First determination of azole resistance in *Aspergillus fumigatus* strains carrying the TR34/L98H mutations in Turkey. J. Infect. Chemother..

[72] Rath P.M., Buchheidt D., Spiess B., Arfanis E., Buer J., Steinmann J. (2012). First reported case of azole-resistant *Aspergillus fumigatus* due to the TR34/L98H mutation in Germany. Antimicrob. Agents Chemother..

[73] Rocchi S., Daguindau E., Grenouillet F., Deconinck E., Bellanger A.P., Garcia-Hermoso D., Bretagne S., Reboux G., Millon L. (2014). Azole-resistant *Aspergillus fumigatus* isolate with the TR34/L98H mutation in both a fungicide-sprayed field and the lung of a hematopoietic stem cell transplant recipient with invasive aspergillosis. J. Clin. Microbiol..

[74] Snelders E., Huis In 't Veld R.A., Rijs A.J., Kema G.H., Melchers W.J., Verweij P.E. (2009). Possible environmental origin of resistance of *Aspergillus fumigatus* to medical triazoles. Appl. Environ. Microbiol..

[75] van Ingen J., van der Lee H.A., Rijs T.A., Zoll J., Leenstra T., Melchers W.J., Verweij P.E. (2015). Azole, polyene and echinocandin MIC distributions for wild-type, TR34/L98H and TR46/Y121F/T289A *Aspergillus fumigatus* isolates in The Netherlands. J. Antimicrob. Chemother..

[76] Vermeulen E., Maertens J., De B.A., Nulens E., Boelens J., Surmont I., Mertens A., Boel A., Lagrou K. (2015). Nationwide surveillance of azole resistance in *Aspergillus* diseases. Antimicrob. Agents Chemother..

[77] Ahmad S., Joseph L., Hagen F., Meis J.F., Khan Z. (2015). Concomitant occurrence of itraconazole-resistant and -susceptible strains of *Aspergillus fumigatus* in routine cultures. J. Antimicrob. Chemother..

[78] Ahmad S., Khan Z., Hagen F., Meis J.F. (2014). Occurrence of triazole-resistant *Aspergillus fumigatus* with TR34/L98H mutations in outdoor and hospital environment in Kuwait. Environ. Res..

[79] Badali H., Vaezi A., Haghani I., Yazdanparast S.A., Hedayati M.T., Mousavi B., Ansari S., Hagen F., Meis J.F., Chowdhary A. (2013). Environmental study of azole-resistant *Aspergillus fumigatus* with TR34/L98H mutations in the *cyp*51A gene in Iran. Mycoses.

[80] Chowdhary A., Kathuria S., Randhawa H.S., Gaur S.N., Klaassen C.H., Meis J.F. (2012). Isolation of multiple-triazole-resistant *Aspergillus fumigatus* strains carrying the TR34/L98H mutations in the *cyp*51A gene in India. J. Antimicrob. Chemother..

[81] Chowdhary A., Kathuria S., Xu J., Sharma C., Sundar G., Singh P.K., Gaur S.N., Hagen F., Klaassen C.H., Meis J.F. (2012). Clonal expansion and emergence of environmental multiple-triazole-resistant *Aspergillus fumigatus* strains carrying the TR34/L98H mutations in the *cyp*51A gene in India. PLoS ONE.

[82] Chowdhary A., Sharma C., Kathuria S., Hagen F., Meis J.F. (2014). Azole-resistant *Aspergillus fumigatus* with the environmental TR46/Y121F/T289A mutation in India. J. Antimicrob. Chemother..

[83] Liu M., Zeng R., Zhang L., Li D., Lv G., Shen Y., Zheng H., Zhang Q., Zhao J., Zheng N. (2015). Multiple *cyp*51A-based mechanisms identified in azole-resistant isolates of *Aspergillus fumigatus* from China. Antimicrob. Agents Chemother..

[84] Mohammadi F., Hashemi S.J., Zoll J., Melchers W.J., Rafati H., Dehghan P., Rezaie S., Tolooe A., Tamadon Y., van der Lee H.A. (2015). Quantitative analysis of single-nucleotide polymorphism for rapid detection of TR_34_. Antimicrob. Agents Chemother..

[85] Perveen I., Sehar S., Naz I., Ahmed S. Prospective evaluation of azole resistance in *Aspergillus fumigatus* clinical isolates in Pakistan. Proceedings of 7th Advances Against Aspergillosis.

[86] Seyedmousavi S., Hashemi S.J., Zibafar E., Zoll J., Hedayati M.T., Mouton J.W., Melchers W.J., Verweij P.E. (2013). Azole-resistant *Aspergillus fumigatus*, Iran. Emerg. Infect. Dis..

[87] Wu C.J., Wang H.C., Lee J.C., Lo H.J., Dai C.T., Chou P.H., Ko W.C., Chen Y.C. (2015). Azole-resistant *Aspergillus fumigatus* isolates carrying TR34/L98H mutations in Taiwan. Mycoses.

[88] Le Pape P., Lavergne R.A., Morio F., Alvarez-Moreno C. (2016). Multiple fungicide-driven alterations in azole-resistant *Aspergillus fumigatus*, Colombia, 2015. Emerg. Infect. Dis..

[89] Wiederhold N.P., Garcia-Gil V., Lindner J.R., Sanders C., Fan H., Sutton D.A., Fothergill A.W. Evaluation of *cyp*5A mechanisms of azole resistance in *Aspergillus fumigatus* isolates from the United States. Proceedings of the 7th Trends in Medical Mycology.

[90] Chowdhary A., Sharma C., van den Boom M., Yntema J.B., Hagen F., Verweij P.E., Meis J.F. (2014). Multi-azole-resistant *Aspergillus fumigatus* in the environment in Tanzania. J. Antimicrob. Chemother..

[91] Lavergne R.A., Morio F., Favennec L., Dominique S., Meis J.F., Gargala G., Verweij P.E., Le Pape P. (2015). First description of azole-resistant *Aspergillus fumigatus* due to TR46/Y121F/T289A mutation in France. Antimicrob. Agents Chemother..

[92] Montesinos I., Dodemont M., Lagrou K., Jacobs F., Etienne I., Denis O. (2014). New case of azole-resistant *Aspergillus fumigatus* due to TR46/Y121F/T289A mutation in Belgium. J. Antimicrob. Chemother..

[93] Pelaez T., Monteiro M.C., Garcia-Rubio R., Bouza E., Gomez-Lopez A., Mellado E. (2015). First detection of *Aspergillus fumigatus* azole-resistant strain due to *cyp*51A TR46/Y121F/T289A in an azole-naive patient in Spain. New Microbes. New Infect..

[94] Vermeulen E., Maertens J., Schoemans H., Lagrou K. (2012). Azole-resistant *Aspergillus fumigatus* due to TR46/Y121F/T289A mutation emerging in Belgium, July 2012. Euro. Surveill.

[95] Hagiwara D., Takahashi H., Fujimoto M., Sugahara M., Misawa Y., Gonoi T., Itoyama S., Watanabe A., Kamei K. (2016). Multi-azole resistant *Aspergillus fumigatus* harboring *cyp*51A TR46/Y121F/T289A isolated in Japan. J. Infect. Chemother..

[96] Chen Y., Wang H., Lu Z., Li P., Zhang Q., Jia T., Zhao J., Tian S., Han X., Chen F. (2015). Emergence of TR46/Y121F/T289A in an *Aspergillus fumigatus* isolate from a Chinese patient. Antimicrob Agents Chemother.

[97] Hagiwara D., Takahashi H., Watanabe A., Takahashi-Nakaguchi A., Kawamoto S., Kamei K., Gonoi T. (2014). Whole-genome comparison of *Aspergillus fumigatus* strains serially isolated from patients with aspergillosis. J. Clin. Microbiol..

[98] Denning D.W., Park S., Lass-Florl C., Fraczek M.G., Kirwan M., Gore R., Smith J., Bueid A., Moore C.B., Bowyer P. (2011). High-frequency triazole resistance found in nonculturable *Aspergillus fumigatus* from lungs of patients with chronic fungal disease. Clin. Infect. Dis..

[99] Bueid A., Moore C.B., Denning D.W., Bowyer P. (2013). High-level expression of *cyp*51B in azole-resistant clinical *Aspergillus fumigatus* isolates. J. Antimicrob. Chemother..

[100] Cannon R.D., Lamping E., Holmes A.R., Niimi K., Baret P.V., Keniya M.V., Tanabe K., Niimi M., Goffeau A., Monk B.C. (2009). Efflux-mediated antifungal drug resistance. Clin. Microbiol. Rev..

[101] Chamilos G., Kontoyiannis D.P. (2005). Update on antifungal drug resistance mechanisms of *Aspergillus fumigatus*. Drug Resist. Updat..

[102] Tobin M.B., Peery R.B., Skatrud P.L. (1997). Genes encoding multiple drug resistance-like proteins in *Aspergillus fumigatus* and *Aspergillus flavus*. Gene.

[103] Slaven J.W., Anderson M.J., Sanglard D., Dixon G.K., Bille J., Roberts I.S., Denning D.W. (2002). Increased expression of a novel *Aspergillus fumigatus* ABC transporter gene, *atrF*, in the presence of itraconazole in an itraconazole resistant clinical isolate. Fungal. Genet. Biol..

[104] Rajendran R., Mowat E., McCulloch E., Lappin D.F., Jones B., Lang S., Majithiya J.B., Warn P., Williams C., Ramage G. (2011). Azole resistance of *Aspergillus fumigatus* biofilms is partly associated with efflux pump activity. Antimicrob. Agents Chemother..

[105] da Silva Ferreira M.E., Malavazi I., Savoldi M., Brakhage A.A., Goldman M.H., Kim H.S., Nierman W.C., Goldman G.H. (2006). Transcriptome analysis of *Aspergillus fumigatus* exposed to voriconazole. Curr. Genet..

[106] Fraczek M.G., Bromley M., Buied A., Moore C.B., Rajendran R., Rautemaa R., Ramage G., Denning D.W., Bowyer P. (2013). The *cdr*1B efflux transporter is associated with non-*cyp*51A-mediated itraconazole resistance in *Aspergillus fumigatus*. J. Antimicrob. Chemother..

[107] Xiong Q., Hassan S.A., Wilson W.K., Han X.Y., May G.S., Tarrand J.J., Matsuda S.P. (2005). Cholesterol import by *Aspergillus fumigatus* and its influence on antifungal potency of sterol biosynthesis inhibitors. Antimicrob. Agents Chemother..

[108] Willger S.D., Puttikamonkul S., Kim K.H., Burritt J.B., Grahl N., Metzler L.J., Barbuch R., Bard M., Lawrence C.B., Cramer R.A. (2008). A sterol-regulatory element binding protein is required for cell polarity, hypoxia adaptation, azole drug resistance, and virulence in *Aspergillus fumigatus*. PLoS Pathog..

[109] Blatzer M., Barker B.M., Willger S.D., Beckmann N., Blosser S.J., Cornish E.J., Mazurie A., Grahl N., Haas H., Cramer R.A. (2011). SREBP coordinates iron and ergosterol homeostasis to mediate triazole drug and hypoxia responses in the human fungal pathogen *Aspergillus fumigatus*. PLoS Genet..

[110] Blosser S.J., Cramer R.A. (2012). SREBP-dependent triazole susceptibility in *Aspergillus fumigatus* is mediated through direct transcriptional regulation of *erg*11A (*cyp*51A). Antimicrob. Agents Chemother..

[111] Lamoth F., Juvvadi P.R., Fortwendel J.R., Steinbach W.J. (2012). Heat shock protein 90 is required for conidiation and cell wall integrity in *Aspergillus fumigatus*. Eukaryot. Cell.

[112] Cowen L.E., Lindquist S. (2005). Hsp90 potentiates the rapid evolution of new traits: Drug resistance in diverse fungi. Science.

[113] Camps S.M., Dutilh B.E., Arendrup M.C., Rijs A.J., Snelders E., Huynen M.A., Verweij P.E., Melchers W.J. (2012). Discovery of a *hapE* mutation that causes azole resistance in *Aspergillus fumigatus* through whole genome sequencing and sexual crossing. PLoS ONE.

[114] Lescar J., Meyer I., Akshita K., Srinivasaraghavan K., Verma C., Palous M., Mazier D., Datry A., Fekkar A. (2014). *Aspergillus fumigatus* harbouring the sole Y121F mutation shows decreased susceptibility to voriconazole but maintained susceptibility to itraconazole and posaconazole. J. Antimicrob. Chemother..

[115] Arabatzis M., Kambouris M., Kyprianou M., Chrysaki A., Foustoukou M., Kanellopoulou M., Kondyli L., Kouppari G., Koutsia-Karouzou C., Lebessi E. (2011). Polyphasic identification and susceptibility to seven antifungals of 102 *Aspergillus* isolates recovered from immunocompromised hosts in Greece. Antimicrob. Agents Chemother..

[116] Ziolkowska G., Tokarzewski S., Nowakiewicz A. (2014). Drug resistance of *Aspergillus fumigatus* strains isolated from flocks of domestic geese in Poland. Poult. Sci..

[117] Araujo R., Pina-Vaz C., Rodrigues A.G. (2007). Susceptibility of environmental versus clinical strains of pathogenic *Aspergillus*. Int. J. Antimicrob. Agents.

[118] Sharma C., Hagen F., Moroti R., Meis J.F., Chowdhary A. (2015). Triazole-resistant *Aspergillus fumigatus* harbouring G54 mutation: Is it de novo or environmentally acquired?. J. Glob. Antimicrob. Resist..

[119] Alastruey-Izquierdo A., Mellado E., Pelaez T., Peman J., Zapico S., Alvarez M., Rodriguez-Tudela J.L., Cuenca-Estrella M. (2013). Population-based survey of filamentous fungi and antifungal resistance in Spain (FILPOP study). Antimicrob. Agents Chemother..

[120] Chryssanthou E. (1997). In vitro susceptibility of respiratory isolates of *Aspergillus* species to itraconazole and amphotericin B. Acquired resistance to itraconazole. Scand. J. Infect. Dis..

[121] Kikuchi K., Watanabe A., Ito J., Oku Y., Wuren T., Taguchi H., Yarita K., Muraosa Y., Yahiro M., Yaguchi T. (2014). Antifungal susceptibility of *Aspergillus fumigatus* clinical isolates collected from various areas in Japan. J. Infect. Chemother..

[122] Baddley J.W., Marr K.A., Andes D.R., Walsh T.J., Kauffman C.A., Kontoyiannis D.P., Ito J.I., Balajee S.A., Pappas P.G., Moser S.A. (2009). Patterns of susceptibility of *Aspergillus* isolates recovered from patients enrolled in the transplant-associated infection surveillance network. J. Clin. Microbiol..

[123] Lockhart S.R., Frade J.P., Etienne K.A., Pfaller M.A., Diekema D.J., Balajee S.A. (2011). Azole resistance in *Aspergillus fumigatus* isolates from the ARTEMIS global surveillance study is primarily due to the TR34/L98H mutation in the *cyp*51A gene. Antimicrob. Agents Chemother..

[124] Castanheira M., Messer S.A., Rhomberg P.R., Pfaller M.A. (2016). Antifungal susceptibility patterns of a global collection of fungal isolates: Results of the sentry antifungal surveillance program (2013). Diagn Microbiol Infect. Dis.

[125] Hsueh P.R., Lau Y.J., Chuang Y.C., Wan J.H., Huang W.K., Shyr J.M., Yan J.J., Yu K.W., Wu J.J., Ko W.C. (2005). Antifungal susceptibilities of clinical isolates of *Candida* species, *Cryptococcus neoformans*, and *Aspergillus* species from Taiwan: Surveillance of multicenter antimicrobial resistance in Taiwan program data from 2003. Antimicrob. Agents Chemother..

[126] Asano M., Kano R., Makimura K., Hasegawa A., Kamata H. (2011). Molecular typing and in vitro activity of azoles against clinical isolates of *Aspergillus fumigatus* and *A. niger* in Japan. J. Infect. Chemother..

[127] Kano R., Kohata E., Tateishi A., Murayama S.Y., Hirose D., Shibata Y., Kosuge Y., Inoue H., Kamata H., Hasegawa A. (2015). Does farm fungicide use induce azole resistance in *Aspergillus fumigatus*?. Med. Mycol..

[128] European Commision Health & Consumer Protection Directorate-General Opinion on azole antimycotic resistance. http://ec.europa.eu/food/fs/sc/ssc/out278_en.pdf.

[129] Lockhart S.R. Azole resistance in the Americas: Not catching up with europe (yet). Proceedings of the 7th Trends in Medical Mycology.

[130] Richardson M., Lass-Florl C. (2008). Changing epidemiology of systemic fungal infections. Clin. Microbiol. Infect..

[131] Balajee S.A., Kano R., Baddley J.W., Moser S.A., Marr K.A., Alexander B.D., Andes D., Kontoyiannis D.P., Perrone G., Peterson S. (2009). Molecular identification of *Aspergillus* species collected for the transplant-associated infection surveillance network. J. Clin. Microbiol..

[132] Alastruey-Izquierdo A., Alcazar-Fuoli L., Cuenca-Estrella M. (2014). Antifungal susceptibility profile of cryptic species of *Aspergillus*. Mycopathologia.

[133] Alcazar-Fuoli L., Mellado E., Alastruey-Izquierdo A., Cuenca-Estrella M., Rodriguez-Tudela J.L. (2008). *Aspergillus* section *fumigati*: Antifungal susceptibility patterns and sequence-based identification. Antimicrob. Agents Chemother..

[134] Balajee S.A., Gribskov J., Brandt M., Ito J., Fothergill A., Marr K.A. (2005). Mistaken identity: *Neosartorya pseudofischeri* and its anamorph masquerading as *Aspergillus fumigatus*. J. Clin. Microbiol..

[135] Montenegro G., Sanchez P.S., Jewtuchowicz V.M., Pinoni M.V., Relloso S., Temporitti E., Iovannitti C.A., Mujica M.T. (2009). Phenotypic and genotypic characterization of *Aspergillus lentulus* and *Aspergillus fumigatus* isolates in a patient with probable invasive aspergillosis. J. Med. Microbiol..

[136] Sugui J.A., Vinh D.C., Nardone G., Shea Y.R., Chang Y.C., Zelazny A.M., Marr K.A., Holland S.M., Kwon-Chung K.J. (2010). *Neosartorya udagawae* (*Aspergillus udagawae*), an emerging agent of aspergillosis: How different is it from *Aspergillus fumigatus*?. J. Clin. Microbiol..

[137] Vinh D.C., Shea Y.R., Jones P.A., Freeman A.F., Zelazny A., Holland S.M. (2009). Chronic invasive aspergillosis caused by *Aspergillus viridinutans*. Emerg. Infect. Dis..

[138] Hendrickx M., Beguin H., Detandt M. (2012). Genetic re-identification and antifungal susceptibility testing of *Aspergillus* section *nigri* strains of the BCCM/IHEM collection. Mycoses.

[139] Stevens D.A., Espiritu M., Parmar R. (2004). Paradoxical effect of caspofungin: Reduced activity against *Candida albicans* at high drug concentrations. Antimicrob Agents Chemother.

[140] Alcazar-Fuoli L., Mellado E., Alastruey-Izquierdo A., Cuenca-Estrella M., Rodriguez-Tudela J.L. (2009). Species identification and antifungal susceptibility patterns of species belonging to *Aspergillus* section *nigri*. Antimicrob. Agents Chemother..

[141] Szigeti G., Kocsube S., Doczi I., Bereczki L., Vagvolgyi C., Varga J. (2012). Molecular identification and antifungal susceptibilities of black *Aspergillus* isolates from otomycosis cases in Hungary. Mycopathologia.

[142] Krishnan S., Manavathu E.K., Chandrasekar P.H. (2009). *Aspergillus flavus*: An emerging non-*fumigatus Aspergillus* species of significance. Mycoses.

[143] Balajee S.A., Lindsley M.D., Iqbal N., Ito J., Pappas P.G., Brandt M.E. (2007). Nonsporulating clinical isolate identified as *Petromyces alliaceus* (anamorph *Aspergillus alliaceus*) by morphological and sequence-based methods. J. Clin. Microbiol..

[144] Ozhak-Baysan B., Alastruey-Izquierdo A., Saba R., Ogunc D., Ongut G., Timuragaoglu A., Arslan G., Cuenca-Estrella M., Rodriguez-Tudela J.L. (2010). *Aspergillus alliaceus* and *Aspergillus flavus* co-infection in an acute myeloid leukemia patient. Med. Mycol..

[145] Liu W., Sun Y., Chen W., Liu W., Wan Z., Bu D., Li R. (2012). The T788G mutation in the *cyp*51C gene confers voriconazole resistance in *Aspergillus flavus* causing aspergillosis. Antimicrob. Agents Chemother..

[146] Paul R.A., Rudramurthy S.M., Meis J.F., Mouton J.W., Chakrabarti A. (2015). A novel Y319H substitution in *cyp*51C associated with azole resistance in *Aspergillus flavus*. Antimicrob Agents Chemother.

[147] Lass-Florl C., Griff K., Mayr A., Petzer A., Gastl G., Bonatti H., Freund M., Kropshofer G., Dierich M.P., Nachbaur D. (2005). Epidemiology and outcome of infections due to *Aspergillus terreus*: 10-year single centre experience. Br. J. Haematol..

[148] Steinbach W.J., Perfect J.R., Schell W.A., Walsh T.J., Benjamin D.K. (2004). In vitro analyses, animal models, and 60 clinical cases of invasive *Aspergillus terreus* infection. Antimicrob. Agents Chemother..

[149] Graybill J.R., Hernandez S., Bocanegra R., Najvar L.K. (2004). Antifungal therapy of murine *Aspergillus terreus* infection. Antimicrob. Agents Chemother..

[150] Arendrup M.C., Jensen R.H., Grif K., Skov M., Pressler T., Johansen H.K., Lass-Florl C. (2012). In vivo emergence of *Aspergillus terreus* with reduced azole susceptibility and a *cyp*51A M217I alteration. J. Infect. Dis..

[151] Samson R.A., Peterson S.W., Frisvad J.C., Varga J. (2011). New species in *Aspergillus* section *terrei*. Stud. Mycol..

[152] Guinea J., Sandoval-Denis M., Escribano P., Pelaez T., Guarro J., Bouza E. (2015). *Aspergillus citrinoterreus*, a new species of section *terrei* isolated from samples of patients with nonhematological predisposing conditions. J. Clin. Microbiol..

[153] Kathuria S., Sharma C., Singh P.K., Agarwal P., Agarwal K., Hagen F., Meis J.F., Chowdhary A. (2015). Molecular epidemiology and in vitro antifungal susceptibility of *Aspergillus terreus* species complex isolates in Delhi, India: Evidence of genetic diversity by amplified fragment length polymorphism and microsatellite typing. PLoS ONE.

[154] Varga J., Houbraken J., van der Lee H.A., Verweij P.E., Samson R.A. (2008). *Aspergillus calidoustus* sp. nov., causative agent of human infections previously assigned to *Aspergillus ustus*. Eukaryot. Cell.

[155] Alastruey-Izquierdo A., Cuesta I., Houbraken J., Cuenca-Estrella M., Monzon A., Rodriguez-Tudela J.L. (2010). In vitro activity of nine antifungal agents against clinical isolates of *Aspergillus calidoustus*. Med. Mycol..

[156] Verweij P.E., Ananda-Rajah M., Andes D., Arendrup M.C., Bruggemann R.J., Chowdhary A., Cornely O.A., Denning D.W., Groll A.H., Izumikawa K. (2015). International expert opinion on the management of infection caused by azole-resistant *Aspergillus fumigatus*. Drug Resist. Updat..

[157] Seyedmousavi S., Melchers W.J., Mouton J.W., Verweij P.E. (2013). Pharmacodynamics and dose-response relationships of liposomal amphotericin B against different azole-resistant *Aspergillus fumigatus* isolates in a murine model of disseminated aspergillosis. Antimicrob. Agents Chemother..

[158] Mukherjee P.K., Sheehan D.J., Hitchcock C.A., Ghannoum M.A. (2005). Combination treatment of invasive fungal infections. Clin. Microbiol. Rev..

[159] Krishnan-Natesan S., Wu W., Chandrasekar P.H. (2012). In vitro efficacy of the combination of voriconazole and anidulafungin against voriconazole-resistant *cyp*51A mutants of *Aspergillus fumigatus*. Diagn. Microbiol. Infect. Dis..

[160] Marr K.A., Schlamm H.T., Herbrecht R., Rottinghaus S.T., Bow E.J., Cornely O.A., Heinz W.J., Jagannatha S., Koh L.P., Kontoyiannis D.P. (2015). Combination antifungal therapy for invasive aspergillosis: A randomized trial. Ann. Intern. Med..

[161] Seyedmousavi S., Bruggemann R.J., Melchers W.J., Verweij P.E., Mouton J.W. (2013). Pharmacodynamics of anidulafungin against clinical *Aspergillus fumigatus* isolates in a nonneutropenic murine model of disseminated aspergillosis. Antimicrob. Agents Chemother.

[162] Denning D.W., Bowyer P. (2013). Voriconazole resistance in *Aspergillus fumigatus*: Should we be concerned?. Clin. Infect. Dis..

[163] European Centre for Disease Prevention and Control Risk assessment on the impact of environmental usage of triazoles on the development and spread of resistance to medical triazoles in *Aspergillus* species. http://ecdc.europa.eu/en/publications/Publications/risk-assessment-impact-environmental-usage-of-triazoles-on-Aspergillus-spp-resistance-to-medical-triazoles.pdf.

